# Supplementary material for: A fault-intrusion-tolerant system and deadline-aware algorithm for scheduling scientific workflow in the cloud
Source: PeerJ Comput Sci. 2021 Nov 2;7:e747. doi: 10.7717/peerj-cs.747 (PMC8576554; doi:10.7717/peerj-cs.747)
Supplement: Supplemental Information 6 [file peerj-cs-07-747-s006.zip › Myproject1.1/docs/org/cloudbus/cloudsim/power/models/PowerModel.html]

PowerModel


JavaScript is disabled on your browser.


- Overview
- Package
- Class
- Use
- Tree
- Deprecated
- Index
- Help

- Prev Class
- Next Class

- Frames
- No Frames

- All Classes

- Summary:
- Nested |
- Field |
- Constr |
- Method

- Detail:
- Field |
- Constr |
- Method


org.cloudbus.cloudsim.power.models

## Interface PowerModel

- All Known Implementing Classes:
  :   PowerModelCubic, PowerModelLinear, PowerModelSpecPower, PowerModelSpecPowerHpProLiantMl110G3PentiumD930, PowerModelSpecPowerHpProLiantMl110G4Xeon3040, PowerModelSpecPowerHpProLiantMl110G5Xeon3075, PowerModelSpecPowerIbmX3250XeonX3470, PowerModelSpecPowerIbmX3250XeonX3480, PowerModelSpecPowerIbmX3550XeonX5670, PowerModelSpecPowerIbmX3550XeonX5675, PowerModelSqrt, PowerModelSquare

  ---

    

  ```
  public interface PowerModel
  ```

  The PowerModel interface needs to be implemented in order to provide a model of power consumption
  depending on utilization for system components.
  If you are using any algorithms, policies or workload included in the power package, please cite
  the following paper:
  Anton Beloglazov, and Rajkumar Buyya, "Optimal Online Deterministic Algorithms and Adaptive
  Heuristics for Energy and Performance Efficient Dynamic Consolidation of Virtual Machines in
  Cloud Data Centers", Concurrency and Computation: Practice and Experience (CCPE), Volume 24,
  Issue 13, Pages: 1397-1420, John Wiley & Sons, Ltd, New York, USA, 2012

  Since:
  :   CloudSim Toolkit 2.0

- - ### Method Summary

    Methods

    | Modifier and Type | Method and Description |
    | --- | --- |
    | `double` | `getPower(double utilization)` Get power consumption by the utilization percentage according to the power model. |

- - ### Method Detail


    - #### getPower

      ```
      double getPower(double utilization)
                      throws java.lang.IllegalArgumentException
      ```

      Get power consumption by the utilization percentage according to the power model.

      Parameters:
      :   `utilization` - the utilization

      Returns:
      :   power consumption

      Throws:
      :   `java.lang.IllegalArgumentException` - the illegal argument exception


- Overview
- Package
- Class
- Use
- Tree
- Deprecated
- Index
- Help

- Prev Class
- Next Class

- Frames
- No Frames

- All Classes

- Summary:
- Nested |
- Field |
- Constr |
- Method

- Detail:
- Field |
- Constr |
- Method
